# The Impact of Dietary Protein in Complementary Foods on Infant Growth and Body Composition in a Population Facing the Double Burden of Malnutrition: Protocol for a Multicenter, Prospective Cohort Study

**DOI:** 10.2196/18112

**Published:** 2020-09-17

**Authors:** Kulnipa Kittisakmontri, Julie Lanigan, Jonathan C K Wells, Mary Fewtrell

**Affiliations:** 1 Childhood Nutrition Research Centre, Population Policy and Practice Research and Teaching Department Great Ormond Street Institute of Child Health University College London London United Kingdom; 2 Department of Pediatrics Faculty of Medicine Chiang Mai University Chiang Mai Thailand

**Keywords:** complementary feeding, protein intake, double burden of malnutrition, infant growth, body composition

## Abstract

**Background:**

Protein is an essential macronutrient with an important role during complementary feeding. Low protein intake contributes to undernutrition while high intake, especially from animal sources, may increase obesity risk. However, the influences of different protein sources (dairy, meat, and plants) on growth, and underlying mechanisms for these effects, are poorly understood. Animal-sourced foods provide both high-quality protein and iron and are recommended to improve iron status. However, it is unclear whether current dietary recommendations are adequate to support healthy growth and optimize iron status. These issues are of particular concern in countries facing the double burden of malnutrition, the coexistence of all forms of malnutrition. More evidence is needed to develop appropriate recommendations for these countries.

**Objective:**

This study will investigate associations between protein intake during complementary feeding and growth, body composition, and iron status of infants in Thailand, a country facing the double burden of malnutrition. The study will also explore how different protein sources influence growth via the growth hormone—insulin-like growth factor I (IGF-1) axis and plasma amino acids.

**Methods:**

A multicenter cohort study will be conducted in Chiang Mai, Thailand, in 150 healthy term infants aged 4-6 months with birth weight ≥2500 g. Demographic data, dietary intake, and anthropometry will be collected at 6, 9, and 12 months. Dietary intake will be assessed using 24-hour dietary recalls, 3-day food records, and food frequency questionnaires. Blood samples for iron status, growth hormone, IGF-1, insulin-like growth factor-binding protein III (IGFBP-3), and plasma amino acids and urine samples for body composition analysis using stable isotope dilution will be obtained at 12 months.

**Results:**

The recruitment of study participants and data collection was undertaken from June 2018 to May 2019. Data and laboratory analyses are ongoing and are expected to be completed by December 2020. A total of 150 participants were enrolled, and 146 completed the study. We hypothesized that protein intake from animal-sourced foods in recommended quantities could support normal weight and length gain and lower the risk of undernutrition associated with similar amounts of plant-based protein. However, higher protein intake, especially from milk protein, may be linked to increased body fat via plasma amino acids and the growth hormone-IGF axis.

**Conclusions:**

The results of this study will provide data on current complementary feeding practices, focusing on protein and iron intake in Thai infants. This information, combined with data on associations with infant growth and iron status, will help inform complementary feeding recommendations for this population and may be found relevant to other settings experiencing the double burden of malnutrition.

**International Registered Report Identifier (IRRID):**

DERR1-10.2196/18112

## Introduction

In 2016, while 52 and 155 million children around the globe were suffering from wasting and stunting, 41 million children under the age of 5 years were overweight or obese [1]. Therefore, the United Nations General Assembly launched a new policy, the United Nations Decade of Action on Nutrition from 2016 to 2025. The policy aims to end hunger and eradicate all forms of malnutrition worldwide [1]. In this proclamation, the double burden of malnutrition, the coexistence of undernutrition, overweight, obesity, and diet-related noncommunicable diseases are highlighted as an emerging public health problem in many countries experiencing socioeconomic transition. In order to achieve the goals of the proclamation, early life nutrition, especially during the first 1000 days of life, has been identified as one of the primary targets of intervention [2].

Along with optimal maternal nutrition and exclusive breastfeeding until 6 months of age, complementary feeding plays a pivotal role in early life nutrition from the age of 6 to 24 months. This period is also the most challenging, as a systematic review indicated that no single intervention can optimize growth outcomes for infants living in different circumstances with variations in the availability of micronutrient-rich foods, degree of household food insecurity, and traditional complementary feeding practices [3]. Among various topics related to complementary feeding, dietary protein is interesting as it is linked to all forms of malnutrition.

Protein is an essential macronutrient needed to build organ systems and body structures and mediates many physiological functions throughout life. For infants and young children, protein requirements per kg body weight are higher than any other age groups due to their rapid growth [4]. In addition, the proportion of energy from protein abruptly changes from 5% to around 15% of total energy intake when breastfed infants are introduced to complementary foods [5]. According to current evidence, insufficient protein intake during complementary feeding can contribute to undernutrition [6-8], whereas too much may increase the risk of overweight and obesity in later life [9-13].

Nevertheless, it is not only the amount of dietary protein that matters but also the source. Dietary protein comes from either animals or plants. Some studies have shown a link between protein sources and growth via plasma amino acids and the growth hormone—insulin-like growth factor I (IGF-1) axis. Semba et al demonstrated that stunted children had lower levels of plasma essential amino acids [14]. In contrast, a multicenter randomized controlled trial in 5 European countries showed that plasma Leucine and IGF-1 were positively associated with body mass index z-score (BMIZ) in infants who received high protein intake from infant formula [15]. These findings have two important implications. First, protein from animal-sourced foods such as milk, meat, and eggs provides all essential amino acids, while plant-based proteins (cereals, legumes, vegetables) usually lack one or two essential amino acids. Thus, it can be assumed that the effect of dietary protein on growth is more closely related to protein source than quantity. Second, as the proportion of leucine in the amino acid content of whey is higher in milk than that in meat (14% vs 8%, respectively) [16], protein intake from nondairy animal-sourced foods may not have the same influence on BMIZ as similar intake from milk. However, few studies have measured body composition as it relates to the intake of different protein sources.

Most evidence comes from two separate paradigms. The first has focused on lower- and middle-income countries where the researchers try to encourage higher protein intake in terms of both quantity and quality to overcome undernutrition, while the second paradigm is emerging in higher-income settings where researchers are aiming to prevent obesity by reducing the protein intake of infants and young children. As a result of these separate approaches, it is difficult to define guidelines for countries that are now facing the double burden of malnutrition, the majority of which are low- to middle-income countries.

Thailand is a country facing the double burden of malnutrition. The latest national and international surveys demonstrate a slowly declining percentage of childhood stunting, a high prevalence of iron deficiency, and rapidly increasing proportions of overweight, obesity, and diet-related noncommunicable diseases at a population level [17-19]. However, there have been no recent studies on protein intake among Thai infants. Although the latest Thai dietary reference intakes recommend a protein intake of 1.56 g/kg/day for Thai infants aged 6-11 months [20], there have been no clinical studies to support the adequacy of this recommendation in Thai infants as the recommendation is adapted from international guidelines [4].

### Aims and Hypotheses

We aim to understand the impact of dietary protein in early life using a holistic approach. This study will fill some of the knowledge gaps related to underlying mechanisms through the following objectives and hypotheses:

1. We will investigate the associations between the amounts and sources of dietary protein typically provided to Thai infants aged 6 to 12 months and changes in growth, including weight-for-age z-score (WAZ), weight-for-length z-score (WLZ), length-for-age z-score (LAZ), and BMIZ over 6-12 months. We will test the hypothesis that protein intake from animal-sourced foods in recommended quantities can support normal weight and length gain and reduce the risk of undernutrition associated with similar amounts of plant-based protein.
2. We will investigate the associations between the amounts and sources of dietary protein typically provided to Thai infants aged 6 to 12 months and body composition at 12 months of age. We will test the hypothesis that a higher intake of protein from animal-sourced foods, especially dairy protein, is associated with an increased proportion of body fat during infancy.
3. We will investigate the associations between complementary foods that provide different amounts of protein from animal-sourced foods and plant-based diets typically provided to Thai infants aged 6 to 12 months with iron status at 12 months of age. We will test the hypothesis that the timely introduction of protein from animal-sourced foods, especially red meat in the recommended amount, is associated with improved iron status and lower risk of iron deficiency anemia, without increasing the risk of overweight/obesity.
4. We will investigate the possible mechanisms linking dietary protein and growth in early life through metabolomic processes. We will test the hypothesis that high protein intake from animal-sourced foods is associated with increased plasma essential amino acids, which may be correlated with concentrations of growth hormone, IGF-1, and IGFBP-3.

## Methods

### Study Design and Approval

#### Study Design

This protocol is for a multicenter, prospective cohort study.

#### Study Setting

This cohort study will be performed in three well-baby clinics in Chiang Mai, Thailand. We selected these well-baby clinics based on their locations and opening times that did not overlap, allowing the single investigator to attend all clinic sessions. Two study sites are in urban areas, and another is in a suburban area. One clinic in the urban area is in a maternal and child-friendly hospital where infant formula is prohibited, and the breastfeeding rate is higher than the average for other Thai hospitals.

#### Ethics Approval

The study will be conducted in accordance with the Declaration of Helsinki. Participation is voluntary, and subjects can withdraw from the study at any stage. The study enrolment is completed when written informed consent is obtained from the legal guardian. Identifiable data of participants and their families will be kept in a secure location, only accessible to the researchers. Ethics approvals have been obtained from the University College London Ethics committee, United Kingdom (Approval ID: 12551/001), and the Ethics Committee of the Faculty of Medicine, Chiang Mai University, Thailand (Approval ID: PED-2561-05287).

### Definitions

Key terms are defined in Textbox 1.

**Definitions.**

*Complementary feeding* is all solid and liquid foods other than breast milk or infant and follow-on formula, as suggested by the European Food Safety Authority (EFSA) [21] and the position papers from the European Society for Pediatric Gastroenterology Hepatology and Nutrition [22].

*Stunting* is defined as LAZ <2 SD below the median of the WHO growth standards for children aged under 5 years [23].

*Wasting* is defined as WLZ <2 SD below the median of the WHO child growth standards for children aged under 5 years [23].

*Underweight* is defined as WAZ is <2 SD below the median of the WHO child growth standards for children aged under 5 years [23].

*Overweight* is defined as WLZ >2 SD above the median of the WHO child growth standards for children aged under 5 years [24].

*Obesity* is defined as WLZ >3 SD above the median of the WHO child growth standards for children aged under 5 years [24].

*Iron deficiency* is defined by at least one of the following serum ferritin less than 12 μg/L (if erythrocyte sedimentation rate, ESR ≤10 mm/h) or serum ferritin less than 30 μg/L (if ESR >10 mm/h) or serum transferrin saturation less than 16% [25].

*Iron deficiency anemia* is defined as iron deficiency plus hemoglobin <11.0 g/dL [25].

*Exclusive breastfeeding* means the infant receives only breast milk without anything else except for water and necessary medication.

*Predominant breastfeeding* means that breast milk is more than 50% of daily milk intake after 6 months of age.

*Predominant formula feeding* means that infant or follow-on formula provides more than 50% of daily milk intake and for a longer period than breastfeeding.

*Animal-sourced food* is defined as any food that is edible and originates from animals such as milk, dairy products, meat (ie, beef, pork, poultry), processed meats, fishes, insects, and eggs.

*Plant-based protein* is defined as dietary proteins that are edible and originate from plants such as cereals, lentils, legumes, nuts, and vegetables.

The *primary caregiver* is defined as the person who spends the most time looking after the infant on a daily basis.

### Study Population

#### Inclusion Criteria

Eligible infants are full-term (gestational age ≥37 weeks), healthy, singleton infants aged 4-6 months whose birth weight was ≥2500 g.

#### Exclusion Criteria

Infants with any underlying or chronic diseases, those with a known case of, or recovery from, protein-energy malnutrition, and infants regularly receiving medication except mineral and vitamin supplementation will be excluded.

### Participant Selection and Recruitment

Parents or caregivers of infants aged 4-6 months who are eligible for the study will be approached by a researcher when they routinely visit the well-baby clinic and asked if they are interested in taking part in the study. The researcher will give the parent an information sheet and answer all queries on a case by case basis. If they consider participating, contact details will be obtained. After 48 hours, the researcher will call the parents to ask if they wish to take part and, if they agree, the first appointment will be scheduled. At the first visit, informed consent will be signed by the parent or legal guardian who is the primary caregiver of the participant.

### Data Collection

There are 3 visits at the well-baby clinic for each participant when they are 6, 9, and 12 months of age (Table 1).

**Table 1:** Data and collection of biological samples.

Data collection	First visit, age 6 months	Second visit, age 9 months	Third visit, age 12 months
Demographic data	Family type, family income (monthly), primary caregiver Parental education and occupation Parental body weight and height Prenatal screening and problems Mode of delivery, gestational age at birth, birth weight, length, and head circumference Postnatal problems (eg, neonatal jaundice)	N/A^a^	N/A
Milk feeding	Type of milk feeding, duration of exclusive breastfeeding	Type of milk feeding, duration of breastfeeding/using breast milk	Type of milk feeding, duration of breastfeeding/using breast milk
Dietary intake	Age of first introduction of complementary feeding, type of first complementary feeding, age of introducing each type of diet (cereal, meat, egg, milk) 24-hour dietary recall Food Frequency and Baby Eating Behavior Questionnaire	Age of introducing each type of diet 24-hour dietary recall 3-day food record Food Frequency and Baby Eating Behavior Questionnaire	Age of introducing each type of diet 24-hour dietary recall 3-day food record Food Frequency and Baby Eating Behavior Questionnaire
Anthropometric measurements	Bodyweight, length, head circumference, mid-upper arm circumference	Bodyweight, length, head circumference, mid-upper arm circumference	Bodyweight, length, head circumference, mid-upper arm circumference
Biological sample collection	N/A	N/A	Blood sample: 5 ml venous blood Body composition: 1.5 mL urine × 3 samples, before and after dosing with Deuterium oxide
Other information	Feeding problems, acute illness	Feeding problems, acute illness	Feeding problems, acute illness

^a^N/A: not applicable.

### Biological Sample Collection, Preparation, and Storage

#### Blood Samples

At 12 months of age, a venous blood sample will be obtained by experienced nurses or health professionals. Five milliliters of venous blood will be separated into 4 collecting tubes; 1 EDTA tube for ESR and plasma amino acids, 1 EDTA microtainer for complete blood count (CBC), 1 clotted blood tube for growth hormone, IGF-1, IGFBP-3, and 1 heparinized microtainer for serum ferritin, serum iron, and serum total iron-binding capacity. The blood samples for CBC, ESR, serum ferritin, serum iron, and total iron-binding capacity will be analyzed on the day of blood collection. The rest of the blood samples for growth hormone, IGF-1, IGF-binding protein 3 (IGFBP-3), and plasma amino acid analysis will be centrifuged and aliquoted into cryovials within the same day and stored at –80^o^C until analysis.

#### Urine Samples

Infant body composition will be measured by the isotope dilution technique using deuterium oxide (^2^H_2_O). At 12 months of age, the dose of deuterium oxide calculated according to infant body weight (0.1 g per kg ^2^H_2_O body weight) will be prepared as a solution (in water, juice, or milk) and orally administered to infants during their final visit. Urine samples will be obtained before dosing and after dosing at 6 and 24 hours. Urine samples will be stored at –80^o^C until analysis.

### Study Outcomes

#### Primary Outcome

The primary outcome measure will be the conditional change of growth parameters (WAZ, WLZ, LAZ) from 6-12 months of age.

#### Secondary Outcomes

Secondary outcome measures will be (1) the conditional change of BMIZ from 6-12 months of age, (2) anthropometric measurements at 12 months of age, (3) infant body composition at 12 months of age, (4) iron status including CBC, serum ferritin, SI and total iron-binding capacity at 12 months of age, (5) growth-related hormones (growth hormone, IGF-1, IGFBP-3) at 12 months of age, and (6) plasma amino acids at 12 months of age.

### Outcome Measures

#### Energy and Nutrient Intake

Dietary data will be collected using 24-hour dietary recalls and the Food Frequency Questionnaire at 6, 9, and 12 months and 3-day food records at 9 and 12 months. All dietary data will be converted to energy and nutrient intake (ie, carbohydrate, fat, protein, calcium, phosphorus, iron, zinc, vitamin B1, vitamin B2, vitamin C, and vitamin A) by using the Institute of Nutrition Mahidol University Calculation program version 4.0 developed by the Institute of Nutrition, Mahidol University, Bangkok, Thailand [26]. This program reports total protein and iron intake from both animal and plant sources. Breastfeeding will be recorded as frequency and duration (minutes per meal) each day, then the volume of breast milk will be estimated using the average amount from two algorithms [27,28].

#### Anthropometric Assessment

Bodyweight will be measured using calibrated electronic scales accurate to 0.01 kg and recumbent length using a standard wood board accurate to 0.1 cm. The SD z-scores for weight-for-age, length-for-age, weight-for-length, and BMI will be calculated using WHO software (WHO Anthro version 3.2.2) based on the 2007 WHO Growth Standard [29]. Although the WHO recommends weight-for-length to be used to determine wasting and overweight for children aged <2 years, a recent study showed that infant BMI had a higher positive predictive value than weight-for-length for childhood obesity [30].Therefore, the present study will measure both indices.

### Dietary Data Collection Tools

The format of the 24-hour dietary recalls and 3-day food records have been adapted from Lanigan et al [27] and translated into the Thai language by the researcher who is a Thai native speaker. The semi-quantitative Food Frequency Questionnaire was developed with information on food items consumed by Thai infants and toddlers aged 0-3 years relating to the food consumption data of Thailand 2016 [31]. The Food Frequency Questionnaire contains 130 food items. Appetite traits will be evaluated by the Baby Eating Behavior Questionnaire, which was developed by Llewellyn et al from the Gemini birth cohort [32].

### Laboratory Analyses

#### Infant Body Composition

Urine samples will be measured by isotope-ratio mass spectrometry (Gasbench-Delta XP system, Thermo Fisher Scientific, Bremen).

#### Iron Status

CBC will be analyzed by using an automated hematological analyzer (Sysmex XN 9103, Sysmex UK). An automated system will be used for the direct determination of ESR (VES-MATIC cube 30, DIESSE Diagnostica Senese). Serum ferritin, serum iron, and total iron-binding capacity will be analyzed by using a chemiluminescent method (Cobas modular analyzer, Roche Diagnostics, F Hoffmann-La Roche, Germany).

#### Growth-Related Hormones

Growth hormone, IGF-1, and IGFBP-3 will be analyzed by enzyme-linked immunosorbent assay (IMMULITE 2000, Siemens Healthcare Diagnostics Products).

#### Plasma Amino Acids

Twenty plasma amino acids will be analyzed by using the high-performance liquid chromatography (Biochrom30 amino acid analyzer, Biochrom).

### Statistical Analysis

There are two main parts of the statistical analysis. First, we will summarize the population characteristics of milk feeding, complementary feeding, and prevalence of undernutrition, overweight/obesity, and iron deficiency. Second, we will examine the associations between protein intake and source during complementary feeding and the outcomes specified in the objectives and hypotheses. All statistical tests will be considered significant when the *P* value is <.05 or 95% CIs of odds ratios do not include 1.

Descriptive data will be presented as mean (SD), median with interquartile range, or percentages depending on data type and distribution. These data will include demographic characteristics of infants and families, breastfeeding rate, age of the first introduction of complementary feeding, details of complementary feeding and energy/nutrient intake at each time point, average growth parameters at each time point, growth trajectory, and the prevalence of malnutrition (including wasting, stunting, overweight/ obesity, iron deficiency, iron deficiency anemia).

Protein intake at each time point, both total and separated by dietary source (ie, dairy protein, nondiary animal-based protein, and plant-based protein), will be considered as primary predictors to examine associations between protein intake and outcomes. To investigate associations between those predictors and continuous outcomes such as growth z-scores, percentage of body fat/fat-free mass, and blood parameters, we will use linear regression and correlation models (Pearson or Spearman correlation) for either univariate or multivariate analysis. For multivariate analysis, covariates will be selected for each of the dependent outcomes from previous research publications and our univariate analyses. Additionally, we intend to apply the directed acyclic graphs identifying covariates to reduce confounders [33]. If the dependent outcomes are categorical data such as nutritional status or iron status, binary or logistic regression analysis will be used as the main models.

The study aims to reduce the bias of baseline body size of individual infants on growth outcomes by using conditional growth. This method captures how an individual child deviates from the average growth trajectory in the population, taking into account overall regression to the mean, and expresses growth acceleration or deceleration in an individual relative to this population growth pattern. For example, if the conditional weight z-score is positive, we can interpret that the infant has shown more rapid weight gain than others in the population who had the same baseline size [34]. Therefore, the conditional change in growth parameters from 6 to 12 months will be calculated using linear regression of weight/length z-scores at 12 months on baseline values at 6 months. Residuals from the model represent the difference between observed and predicted growth over this period.

We will use data from the standard recommendations, previous research, and our own data to determine the most appropriate cut-offs for low or high protein intake. We will then compare the outcomes between these groups.

### Sample Size Calculation

We used the two-different means formulae [35] to calculate the sample size required to detect a 0.5 SD difference in growth parameters (WAZ, WLZ, and LAZ) between infants who receive red meat frequently and less frequently from 6-12 months of age (Textbox 2).

**Sample size calculation.**

- Mean difference 0.5 SD
- SD 1.0 (adopted from Olaya G et al [36])
- Power 80%, significance level .05
- Sample size: 126 participants
- With surplus 15% drop out rate, the final number is 148 participants

## Results

Recruitment commenced in June 2018 and was completed in October 2018. Data collection was completed in May 2019. Laboratory analyses are ongoing and are expected to be complete by December 2020. Initially, 150 infants were enrolled, and only 4 (3%) had dropped out at the end of the data collection period. The results of this study will be presented and disseminated through peer-reviewed journals and academic conferences.

## Discussion

Thailand has highlighted early life nutrition as a window of opportunity to overcome the double burden of malnutrition [37]. Several campaigns from the Thai government have been launched to improve maternal and infant nutrition. Examples include a program that encouraged supplementation of Triferdine, which consists of iron, iodine, and folic to women of child-bearing age, promotion of exclusive breastfeeding until 6 months of age as well as iron supplementation of infants and young children aged 6 months to 2 years [38]. However, there is no specific campaign related to complementary feeding and, to the best of our knowledge, no recent studies in this field.

Although recommendations should ideally be based on experimental data, the lack of recent data on dietary protein intake and growth outcomes in Thai infants precluded the development of a practical intervention that could be tested in a randomized controlled trial in this population. To address this issue, we decided to adopt a sequential approach in 3 consecutive studies, beginning with a descriptive study to gather information on the attitudes, knowledge, and practices related to complementary feeding in Thai families. This study provided a clearer picture of complementary feeding among Thai parents and highlighted interesting findings that were used in the design of the current prospective cohort study [39]. For example, rice porridge and mashed banana were the most common first foods for Thai infants with or without a small amount of egg yolk. Some Thai parents delayed providing animal-sourced foods to their children and had concerns that such foods were difficult to digest and had high allergenicity compared with plant-based proteins.

The second phase of our research consists of the observational study described here, incorporating the findings of our previous study. The aim is to investigate the intake of different protein sources during complementary feeding and associations between protein intake and growth outcomes, body composition and iron status in the same population, as well as to investigate possible mechanisms related to those associations. The results of this study may be used to plan the third phase of our research, testing an intervention to improve growth and iron status in this infant population.

## Abbreviations

BMIZ: body mass index z-score
CBC: complete blood count
DBM: double burden of malnutrition
ESR: erythrocyte sediment rate
IGF-1: insulin-like growth factor I
IGFBP-3: insulin-like growth factor-binding protein III
LAZ: length-for-age z-score
WAZ: weight-for-age z-score
WLZ: weight-for-length z-score
